# Improving healthcare efficiency with coordination between levels of care: orthogeriatrics/Mejora de la eficiencia asistencial con la coordinación interniveles: ortogeriatría

**Published:** 2012-05-29

**Authors:** Marta Alvarez de Arcaya Vitoria, Jose Antonio Veras Sanz, Karmele Varea, María Ariztia Sarratea, Cristina Alderete Díez, Olatz Albizua Uriondo

**Affiliations:** Orthogeriatric Unit of Ricardo Berminghan Hospital, Donostia, Spain; Assessment/Admission Unit, Donostia Hospital, Donostia, Spain; Assessment/Admission Unit, Bidasoa Hospital, Irún, Spain; Orthogeriatric Unit of Ricardo Berminghan Hospital, Donostia, Spain; Orthogeriatric Unit of Ricardo Berminghan Hospital, Donostia, Spain; Assessment/Admission Unit, Donostia Hospital, Donostia, Spain

**Keywords:** hip fractures, orthogeriatrics, inter-level coordination, interdisciplinary, dependence, fractura de cadera, ortogeriatría, coordinación interniveles, interdisciplinariedad, dependencia

## Introduction

Hip fracture (HF) is a serious health problem, the prevalence of which is increasing with population ageing [1, 2]. It commonly causes dependency and has high social and healthcare costs [3].

HF patients often require long stays on trauma wards due to their age [1], other characteristics (multiple pathologies and medications, as well high rates of cognitive impairment and acute complications) and the difficulty of them returning home given their functional deterioration. Further, therapies for functional recovery, indicated in these patients to help them return to their previous level of independence, tend to be applied late, and fail to reach all patients who could potentially make a good recovery; this means social and healthcare costs associated with a level of dependency that could have been avoided.

## Theory and methods

The objectives were to:

- Enable the return to the greatest possible level of independence among all patients with potential to make a good recovery
- Provide care that meets the specific needs of elderly patients with HF
- Ensure that the hospital stay at different levels of care is appropriate
- Tailor the social and healthcare provided on discharge and avoid delays in care between levels.

In May 2010, a comprehensive and interdisciplinary, coordinated care project was launched for patients with HF, with a protocol for referral from the Department of Traumatology of Donostia Hospital (tertiary care) to the Orthogeriatric Unit (OGU) of Ricardo Berminghan Hospital (sub-acute care), this being extended to include Bidasoa Hospital (regional hospital) in September 2010. All patients admitted through the Emergency Department with HF have been included in this project according to the following protocol:

- Day 1 of stay – patient assessment and recommendations for social support.
- Day 5 of stay – after the surgical intervention, a thorough assessment of the patient is carried out, considering their functional status before the fall and at this stage, as well as their mental status, to identify patients who could potentially benefit from care in the Orthogeriatric Unit: those with considerable functional deterioration at the time of assessment [Barthel Index (BI) <60] but who also meet criteria for potentially making a good recovery (BI >30 before the fracture and Spanish version of the Mini-Mental State Examination [MMSE] >10).
- For those transferred to the Orthogeriatric Unit (OGU), during the first 24 hours in the unit therapies for relearning to walk and recovering independence in activities of daily living are initiated and measures taken to stabilise concomitant processes and to prevent common geriatric problems (falls, pressure ulcers, delirium, and iatrogenic complications) following a geriatric model of comprehensive and interdisciplinary assessment and care [4, 5].
- There is planning for discharge from the moment of admission, adapting care provided to the clinical, functional and cognitive status of each patient in coordination with social services. If the patient is considered a candidate for outpatient rehabilitation, access to the gym should be arranged for them before they are discharged to ensure that their physiotherapy is not interrupted.

## Results

In the first year, 390 patients were included in the project, 297 (76%) women and 93 (24%) men. Of these, 238 (61%) were transferred to the OGU, the mean age of this subset being 82.1 years. The mean length of stay of these HF patients in the acute tertiary care hospital fell from 19.7 days in 2009 to 10.6 days in 2010 after the launch of the project.

The functional improvement measured in the OGU was 37.8 points on the BI, the mean BI score on discharge being 72. The mean length of stay in this unit was 21.4 days vs. 34.3 days for HF patients cared for the previous year, with an efficiency score of 1.7 and a Montebello Rehabilitation Factor score of 0.69.

On discharge, 7.3% of patients were institutionalised while for 54 patients (23.1%) their general practitioner was contacted for joint management of their case. Candidates for rehabilitation as outpatients continued their therapy without any interruption on discharge.

## Discussion

This project with the establishment of a new protocol has enabled the early transfer to an Orthogeriatric Unit of all patients who it was judged could potentially benefit from the type of care it offers. This group included individuals with mild-to-moderate cognitive impairment, in whom it has previously been shown that satisfactory recovery of walking ability can be achieved with tailored rehabilitation [6, 7], thereby minimising avoidable dependence.

The use of the different levels of care has improved, with a notable reduction in the length of stay in the acute hospital and also a decrease in the time spent in sub-acute care. This can be attributed to patients being transferred earlier to a care environment focused on the prevention of geriatric problems and recovery of independence, leading to a fall in the number of complications and a lower level of care being required on discharge [8].

This represents considerable cost savings on hospitalisation, as has previously been demonstrated in relation to Orthogeriatric Units of other acute care hospitals [9]. The functional improvements were also good, as has been achieved in other units with similar characteristics [8, 10]: the OGU is efficient in achieving its objective of reducing dependency, managing to enable patients return to a functional status similar to prior to their fall as indicated by the rehabilitation factor score obtained. Further, the coordination between levels of hospital and outpatient care has been strengthened, both in terms of the management of complex cases and of the continuation of physiotherapy on discharge. Importantly, the good functional recovery meant that the rate of institutionalisation on discharge was low [11, 12] and a corresponding saving in the costs of social care services related to hip fractures.

## Conclusions

We found that:

1. A comprehensive assessment of all geriatric patients with hip fracture allowed those individuals who could potentially make a good recovery to be identified.
2. Coordination between levels of care following a protocol enabled a rapid transfer of patients to appropriate types of care avoiding unnecessary delays and hospital stays.
3. The application of a model of geriatric care suited to the needs of these patients led to good functional recovery and a low rate of institutionalisation.
4. The tailoring of the length of stay and type of care provided to such patients had a positive impact on both the direct costs of hospitalisation and those due to the need for care on discharge associated with dependency.

## Conference abstract Spanish

## Introducción

La fractura de cadera (FC) constituye un importante problema de salud cuya prevalencia va en aumento con el envejecimiento poblacional [1, 2], siendo causante de dependencia y ocasionando importantes costes en la atención sanitaria y social [3].

Los pacientes con FC generan con frecuencia estancias prolongadas en los Servicios de Traumatología debido a su edad [1] y características (pluripatología, polifarmacia, prevalencia de deterioro cognitivo, frecuencia de complicaciones agudas) y a la dificultad de retorno al domicilio que su situación de deterioro funcional conlleva. Además las terapias de recuperación funcional, indicadas en estos pacientes para restablecer su autonomía previa, se aplican en general tarde, no abarcando a todos los pacientes potencialmente recuperables con el consecuente gasto sociosanitario que la dependencia evitable genera.

## Teoría y métodos

Con el objetivo de:

- Restablecer la máxima autonomía de todos los pacientes potencialmente recuperables.
- Proporcionar asistencia acorde a las necesidades específicas de los ancianos con FC.
- Adecuar la estancia hospitalaria en los diferentes niveles asistenciales.
- Adaptar los cuidados sociosanitarios al alta y evitar demoras asistenciales interniveles.

En mayo de 2010 se puso en marcha un proyecto de atención coordinada, integral e interdisciplinar a los pacientes afectos de FC, con un protocolo de derivación desde el Servicio de Traumatología del Hospital Donostia (hospital terciario) a la Unidad de Ortogeriatría (UOG) del Hospital Ricardo Berminghan (hospital de subagudos), proyecto al que en septiembre de 2010 se sumó el Hospital del Bidasoa (hospital comarcal).

En el proyecto se han incluido todos los pacientes ingresados de Urgencia con (FC), a los que se aplica el siguiente protocolo:

- Primer día de ingreso, se realiza una valoración y orientación social.
- Al quinto día tras la intervención quirúrgica se realiza una valoración integral del paciente en la que se analiza su situación funcional previa a la caída y en el momento de la valoración y la situación mental, detectando los pacientes que previsiblemente se van a beneficiar de atención en la Unidad de Ortogeriatría: pacientes con deterioro funcional importante en el momento de la valoración: índice de Barthel <de 60 y que cumplan criterios de potencial recuperabilidad: índice de Barthel previo a la fractura >30 y Mini Examen Cognoscitivo MEC>10
- En las primeras 24 horas del ingreso en la Unidad de Ortogeriatría se inician las terapias de reentrenamiento de la marcha y recuperación de autonomía para actividades de la vida diaria, además de estabilización clínica de los procesos coadyuvantes y prevención de síndromes geriátricos (caídas, úlceras por presión (UPPs), síndrome confusional, yatrogenia, etc) siguiendo el modelo geriátrico de valoración/atención integral e interdisciplinar [4, 5].
- El alta se trabaja desde el ingreso adaptando los cuidados necesarios a su situación clínica/funcional y cognitiva en coordinación con trabajo social de base. Si el paciente se considera candidato a proseguir rehabilitación ambulatoria se tramita su entrada en gimnasio previa al alta para no interrumpir el tratamiento fisioterápico.

## Resultados

En el primer año se han incluido en el proyecto 390 pacientes, con una distribución por sexos, 390 (76%) mujeres y 93 (24%) hombres, habiéndose trasladado a la UOG 238 (61%), siendo la edad media de los trasladados de 82,1 años. La estancia media de los pacientes con FC en el Hospital de agudos ha pasado de 19,7 días en 2009 a 10,6 tras inclusión en el proyecto.

La ganancia funcional en la UOG ha sido de 37,8 puntos en el Índice de Barthel (IB), siendo el IB medio al alta de 72 puntos. La estancia media en esta Unidad ha sido de 21,4 días frente a los 34,3 de los pacientes con FC atendidos el año previo, con un índice de eficiencia 1,7 y una eficacia rehabilitadora (Índice de Montebello) de 0,69.

Al alta han sido institucionalizados un 7,3% de los pacientes. En 54 pacientes (23,1%) se contactó con su médico de atención primaria para gestión conjunta del caso. Los candidatos a continuar rehabilitación ambulatoria lo hicieron de forma ininterrumpida tras el alta hospitalaria.

## Discusión

Este proyecto ha posibilitado el paso precoz y protocolizado a la UOG de todos los pacientes que potencialmente se iban a beneficiar de este modelo asistencial, incluyendo aquellos con deterioro cognitivo leve-moderado, en los que ya está demostrada una satisfactoria recuperación de la marcha con programas de rehabilitación adaptados [6, 7] combatiendo con ello la dependencia evitable.

La estancia en los diferentes niveles se ha adecuado con disminución importante en el Hospital de agudos, pero también ha supuesto un descenso en el Hospital de subagudos al ingresar estos pacientes de forma precoz en un entorno de atención orientado a prevención de síndromes geriátricos y recuperación de la autonomía con la consiguiente disminución de complicaciones y necesidad de cuidados al alta que este modelo asistencial conlleva [8]. Esto ha supuesto un importante ahorro en costes de hospitalización como ya se ha demostrado con otras UOG de Hospitales de Agudos [9].

La ganancia funcional ha sido buena al igual que en otras unidades de similares características [8, 10], resultando la UOG eficiente en el alcance de su objetivo de disminución de dependencia, habiendo retornado a los pacientes a una situación funcional cercana a la previa a la caida como lo demuestra la eficacia rehabilitadora alcanzada. Se ha potenciado la coordinación interniveles hospitalarios y extrahospitalarios tanto en la gestión de los casos más complejos como en la continuidad de la fisioterapia en el domicilio. La buena recuperación funcional ha posibilitado una baja institucionalización al alta [11, 12] con el consiguiente ahorro en costes sociales derivados de la fractura de cadera.

## Conclusiones

- La valoración geriátrica integral de todos los pacientes con fractura de cadera ha permitido seleccionar los potencialmente rehabilitables.
- La coordinación interniveles ha permitido el paso adecuado, rápido y protocolizado evitando estancias y demoras innecesarias.
- La aplicación de un modelo de atención geriátrica adecuado a las necesidades de estos pacientes ha posibilitado una buena recuperación funcional y una baja institucionalización.
- La adecuación de estancia y de cuidados ha supuesto un impacto económico positivo tanto en los costes directos de hospitalización como en los resultantes de la necesidad de cuidados al alta que la dependencia genera.

## References

[1] Instituto de información Sanitaria (2010). Estadísticas comentadas: la atención a la fractura de cadera en los hospitales del SNS..

[2] Herrera A, Martínez AA, Fernández L, Gil E, Moreno A (2008). Epidemiology of hip fracture in the elderly in Spain. Bone.

[3] Alarcón Alarcón T, González Montalvo JI (2004). Fractura osteoporótica de cadera. Factores predictivos de recuperación funcional a corto y largo plazo. Anales de Medicina Interna (Madrid).

[4] Sáez López P, Madruga Galán F, Rubio Caballero JA (2007). Detección de problemas en pacientes geriátricos con fractura de cadera. Importancia de la colaboración entre traumatólogo y geriatra. Revista de Ortopedia y Traumatología.

[5] Sáez MP, González Montalvo JI, Alarcón T, Madruga F, Bárcena A (2006). Optimización del tratamiento médico en pacientes geriátricos con fractura de cadera. Influencia del equipo consultor geriátrico. Revista Española de Geriatría y Gerontología.

[6] Heruti RJ, Lusky A, Barell V, Othy A, Adunsky A (1999). Cognitive status at admission: does it affect the rehabilitation outcome of elderly patients with hip fracture?. Archives of Physical Medicine and Rehabilitation.

[7] Goldstein F, Strasser DC, Woodard JL, Roberts VJ (1997). Functional outcomes of cognitively impaired patients on a geriatric rehabilitation unit. Journal of the American Geriatrics Society.

[8] Stenvall M, Olofsson B, Nyberg L, Lundström M, Gustafson Y (2007). Improved performance in activities of daily living and mobility after a multidisciplinary postoperative rehabilitation in older people with femoral neck fracture: a randomized controlled trial with 1-year follow-up. Journal of Rehabilitation Medicine.

[9] González Montalvo JI, Gotor Pérez P, Martín Vega A, Alarcón Alarcón T, Álvarez de Linera JL, Gil Garay E (2011). The acute orthogeriatric unit. Assessment of its effect on the clinical course of patients with hip fractures and an estimate of its financial impact. Revista Española de Geriatría y Gerontología.

[10] Baztán JJ, Fernández-Alonso M, Aguado R, Socorro A (2004). Resultados al año de la rehabilitación tras fractura de fémur proximal en mayores de 84 años. Anales de Medicina Interna.

[11] Parker MJ, Palmer CR (1995). Prediction of rehabilitation alter hip fracture. Age Ageing.

[12] Rosell PA, Parker MJ (2003). Functional outcome after hip fracture. A 1-year prospective outcome study of 275 patients. Injury.

